# Fragility of precision: resistance to mutant p53 reactivation

**DOI:** 10.1038/s41392-026-02830-1

**Published:** 2026-07-13

**Authors:** Andreas C. Joerger, Thorsten Stiewe

**Affiliations:** 1 Institute of Pharmaceutical Chemistry, Goethe University, Frankfurt am Main, Germany; 2 Structural Genomics Consortium (SGC), Buchmann Institute for Molecular Life Sciences, Frankfurt am Main, Germany; 3 Institute of Molecular Oncology, Universities of Giessen and Marburg Lung Center (UGMLC), German Center for Lung Research (DZL), University Marburg, Marburg, Germany; 4 Institute for Lung Health (ILH), Justus Liebig University, Giessen, Germany

**Keywords:** Drug development, Oncogenes, Drug development

In a recent study published in *Cancer Discovery*, Fece de la Cruz et al.^1^ show that cancer patients treated with rezatapopt, a Y220C-selective mutant p53 reactivator, rapidly acquire diverse secondary *TP53* mutations. These findings confirm on-target activity in patients and highlight how the molecular precision of tumor suppressor reactivation creates evolutionary fragility.

**Fig. 1:**
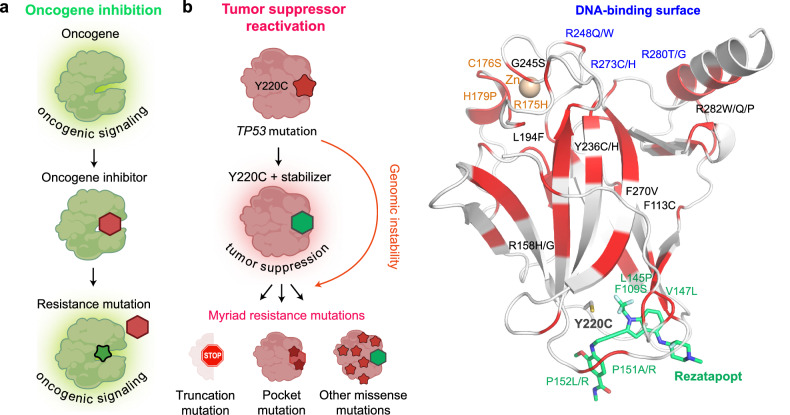
Broader evolutionary escape space in tumor suppressor reactivation than in oncogene inhibition. **a** Targeted therapies against oncogenic drivers inhibit hyperactive proteins generated by activating genetic alterations. Resistance mutations must prevent drug action while preserving oncogenic activity, imposing strong evolutionary constraints and limiting the number of viable escape routes. **b** The small-molecule stabilizer rezatapopt selectively restores the activity of the conformationally unstable p53 cancer mutant Y220C. Tumor cells can escape this reactivation through diverse secondary loss-of-function mutations. This vulnerability may be further amplified by the genomic instability characteristic of many p53-mutant tumors. To illustrate this conundrum, acquired secondary mutations in a patient after rezatapopt treatment reported by Fece de la Cruz et al.^1^ are mapped onto the crystal structure of the Y220C–rezatapopt complex (PDB entry 9S9O). The structure of the Y220C mutant DBD is shown as a cartoon model, with the sites of acquired secondary mutations colored in red and selected mutations labeled. Bound rezatapopt is shown as a green stick model. The spectrum of acquired secondary mutations largely reflects the broad and diverse spectrum of oncogenic mutants observed across cancer patients: DNA-contact mutations (blue labels), mutations directly perturbing the zinc-coordination sphere (orange labels), and mutations that compromise the folding/thermal stability of the DBD, which includes many mutations with an associated temperature-sensitive phenotype (e.g. R158H, L194F, F270V, or R282W). Besides these classical cancer hotspot mutations, some mutations (green labels) affect the rezatapopt binding site and are likely to reduce its binding affinity, in addition to having intrinsic destabilizing effects in some instances. The V147L mutation primarily impairs rezatapopt binding in the Y220C background, while retaining p53 activity when present alone.^1,5^ The figure was prepared using BioRender (www.biorender.com) and PyMOL (www.pymol.org)

Mutations in the *TP53* tumor suppressor gene, encoding the transcription factor p53, occur in approximately half of all human cancers. Yet for decades, mutant p53 was widely considered undruggable. Unlike oncogenic kinases that acquire aberrant enzymatic activity, most cancer-associated p53 mutations abolish transcriptional activation of tumor suppressive programs by destabilizing the DNA-binding domain (DBD), inducing unfolding, or disrupting residues that contact DNA. The therapeutic challenge is therefore fundamentally different: instead of inhibiting abnormal activity, therapies must restore the function of a defective protein, which is a far more difficult task (Fig. 1).

A rare opportunity to overcome this therapeutic impasse emerged from structural studies showing that the frequent cancer mutation Y220C destabilizes the p53 DBD by creating a druggable surface crevice.^2^ This mutation-induced pocket provided a foothold for rational drug design, ultimately leading to the development of rezatapopt, a small-molecule stabilizer that binds the Y220C cavity with high affinity and restores a folded, wild-type-like conformation and transcriptional activity in cancer cells.^3^ Reactivation of p53-dependent gene expression through direct targeting of the conformationally unstable Y220C mutant by chemical chaperones thus provided cellular proof of concept that mutant p53 can be pharmacologically rescued. This strategy has now advanced into the clinic: in a recent phase I study, rezatapopt demonstrated objective responses in patients with advanced solid tumors harboring *TP53* Y220C mutations, presenting compelling clinical evidence that mutant p53 reactivation can produce antitumor activity.^4^

In this context, the recent study by Fece de la Cruz and colleagues provides an important new perspective on the clinical future of this strategy.^1^ Analyzing serial circulating tumor DNA, progression biopsies, and rapid-autopsy material, the authors identified newly acquired secondary *TP53* alterations that emerged during rezatapopt therapy. Strikingly, each of the two patients analyzed developed numerous independent resistance mutations: up to 94 distinct *TP53* variants were detected in circulating tumor DNA in one case. These alterations fell into two broad classes: (i) classical loss-of-function mutations, including missense, nonsense, or frameshift variants; and (ii) mutations that alter the Y220C pocket and may mainly reduce drug binding.

At first glance, this observation mirrors the familiar story of resistance to targeted therapies. Virtually all oncogene inhibitors eventually encounter secondary mutations that prevent drug binding or restore signaling. Yet these findings highlight a deeper challenge that is unique to therapies restoring tumor suppressor function. Targeted therapies against oncogenes inhibit hyperactive proteins that drive tumor growth. Resistance mutations must therefore preserve the oncogenic function while evading drug binding, an evolutionary constraint that drastically limits the number of viable escape routes. By contrast, therapies that attempt to repair tumor suppressors operate under fundamentally different conditions. Here, the drug must restore the function of a structurally fragile protein, whereas the tumor cell can escape by simply breaking it again. From an evolutionary perspective, the barrier to resistance may therefore be dramatically lower (Fig. 1a).

This asymmetry reflects a basic observation in structural biology: destroying protein function is easier than restoring it. This is particularly true for p53, whose intrinsically fragile DBD is highly sensitive to structural perturbation. Systematic mutagenesis studies show that roughly half of all possible missense mutations cause loss of function.^5^ If a drug restores activity by stabilizing a specific mutant, any additional mutation that perturbs the rescued structure may be sufficient to re-establish dysfunction, a vulnerability further amplified by the genomic instability characteristic of many p53-mutant tumors. In the case of rezatapopt, this problem may be particularly pronounced because the drug targets a mutation-specific lesion on the surface. While this exquisite molecular specificity is precisely what makes the compound attractive as a precision therapy, it also creates an inherent vulnerability. Tumor cells can escape p53 reactivation in at least two ways: by altering the geometry of the Y220C binding pocket and preventing drug binding, or by acquiring additional mutations that simply abolish p53 function altogether (Fig. 1b). The evolutionary target space for resistance may therefore be unusually large. Supporting this notion, Dumbrava et al.^4^ reported that one of four patients biopsied at progression acquired inactivating *TP53* mutations (G244D and G245S), consistent with on-target resistance evolution. Nevertheless, it is important to note that the number of patients analyzed for resistance mechanisms remains very limited, so although on-target acquired resistance appears to be a recurrent evolutionary trajectory, it may not be inevitable, and additional or context-dependent escape routes may emerge in larger clinical cohorts.

These considerations do not diminish the importance of rezatapopt. On the contrary, the ability to pharmacologically reactivate mutant p53 represents a remarkable achievement and a milestone in cancer drug discovery. Rather than a setback, these findings offer an instructive glimpse into the evolutionary dynamics that will shape future p53-reactivating therapies.

Several strategies are conceivable that may help address this challenge. One approach could involve developing more potent stabilizers that can accommodate structural variation within the binding pocket, thereby reducing the likelihood that single amino-acid substitutions confer resistance. In addition, combination with allosteric reactivators or small antibody mimetics, such as designed ankyrin repeat proteins (DARPins), which stabilize a broader spectrum of p53 mutants, could potentially increase the evolutionary barrier to resistance. This strategy may be particularly effective in quenching secondary mutations associated with a temperature-sensitive phenotype, as both mutation-induced stability loss and compensatory stabilizing effects are likely to be additive in such a scenario. Alternatively, combination therapies that activate p53 through complementary mechanisms may limit the emergence of resistant clones. However, an important limitation remains: secondary nonsense or frameshift mutations that eliminate p53 expression by nonsense-mediated mRNA decay are likely to represent a universal escape route for any p53-reactivating therapy. In this setting, strategies exploiting vulnerabilities associated with p53 loss-of-function may provide a solution.^1^

The lessons from rezatapopt may extend beyond p53. Efforts to pharmacologically restore tumor suppressor function – through mutant stabilization and folding correction, modulation of degradation pathways, nonsense read-through, or epigenetic reactivation – are gaining momentum. As these strategies move toward the clinic, the evolutionary asymmetry between inhibiting oncogenes and repairing tumor suppressor function may become an important consideration for drug development.

Rezatapopt represents the first successful attempt to resurrect a defective mutant tumor suppressor in the clinic. Resistance mutations highlight both the promise and the fragility of this strategy. Precision enables elegant molecular solutions, but in the evolutionary arms race between therapy and tumor, it may also become the Achilles’ heel that limits long-term efficacy.
